# Health Effects from Secondhand Exposure to E-Cigarettes: A Systematic Review of Peer-Reviewed Articles from 2004–2024

**DOI:** 10.3390/ijerph22091408

**Published:** 2025-09-10

**Authors:** Roengrudee Patanavanich, Chawaphat Thatasawakul, Kamolnut Youngcharoen, Veerapattra Soponvashira, Panpetch Pichetsin

**Affiliations:** Department of Community Medicine, Faculty of Medicine Ramathibodi Hospital, Mahidol University, 270 Rama VI Road, Ratchathewi, Bangkok 10400, Thailand

**Keywords:** e-cigarettes, secondhand, exposure, vaping, passive

## Abstract

**Background**: Since the emergence of e-cigarettes on the market in the early 2000s, the prevalence of e-cigarette use has increased globally. The health risks of using e-cigarettes have been increasingly revealed; however, the health effects on non-users exposed to e-cigarettes are less known. **Methods**: A systematic review was conducted of peer-reviewed articles from 2004 to October 2024 from PubMed and Embase. We focused on the studies that described health outcome measures among non-smokers/vapers exposed to secondhand e-cigarettes. We excluded animal studies and those that did not include human participants. We also omitted studies with financial conflicts of interest with the tobacco industry. **Results**: Of the 8635 studies we found in our search, 16 were included in the final review. Study designs included in our review included a case study, a cohort, eight experimental, four cross-sectional studies, and two observational studies. Health outcome measures were self-reported health symptoms and biomarkers. Ten out of fourteen studies examined respiratory health risks, six described immunological effects, two examined cardiovascular risks, and one explored mental health effects. Self-reported health symptoms such as bronchitis, shortness of breath, asthma, throat irritations, ear infections, and mental health disorders were observed among secondhand e-cigarette exposures when compared with controls. Biomarker measures varied among studies, except for cotinine concentrations of non-smokers/vapers exposed to secondhand e-cigarettes, which were likely to be higher than non-exposed. However, all studies encountered potential limitations. **Conclusions**: Our review found that secondhand e-cigarette exposure is not harmless and may have negative health consequences. However, higher-quality prospective studies remain essential to examine long-term secondhand exposure.

## 1. Introduction

Electronic cigarettes (e-cigarettes) are becoming increasingly popular, particularly among youth [1]. E-cigarettes are available in a range of designs and can be either disposable or reusable. Since their introduction, e-cigarettes have evolved through numerous generations, from early devices that resembled cigarettes to gadgets known as “Toy pods” that use a cartoon-based marketing technique [2]. E-cigarettes are marketed as a healthier alternative to conventional cigarettes as they produce vapor without burning [3]. E-cigarettes work by vaporizing liquid, which typically contains nicotine, flavorings, propylene glycol or glycerol, and other chemicals that help the vaporization process [4]. By 2017, e-cigarettes were available in over 15,000 flavors and flavor combinations [5], and some of them were marketed as safe because their ingredients were approved as food additives, despite the fact that some of them, such as diacetyl, were harmful when inhaled [6]. Moreover, e-cigarettes are promoted as healthier, cheaper, and cleaner than conventional cigarettes; as products that can be smoked anywhere and can be used to avoid smoke-free rules; as devices that do not emit secondhand smoke; and as modern alternatives [4,7]. Compared with adults, adolescents and young adults are more likely to have positive attitudes toward the social and psychological benefits of e-cigarette use [8].

One of the claims that people are generally uncertain about is the health risks of secondhand e-cigarette aerosol (SHA). A survey in 2015 found that one-third of US adults did not know whether SHA caused harm to children, and two-fifths believed it only caused minimal harm [9]. Another survey found that nearly 20% of adolescents felt that SHA was just water vapor [10]. Additionally, a qualitative study in New Zealand found similar perspectives that SHA, its components, and its potential consequences for others were not well understood by e-cigarette users [11]. Knowledge and empirical evidence on SHA are critical for assessing its potential risks and for drafting appropriate policies for vaping in public places.

Studies on the health effects of SHA exposure on humans are limited. A systematic review on the effects of SHA in 2016 [12] included only four studies on direct passive exposure using human volunteers; one was a conference abstract, and three were peer-reviewed papers [13,14,15]. This study aims to systematically review the health risks of SHA on individuals who are directly exposed to it.

## 2. Method

This systematic review followed the Preferred Reporting in Systematic Reviews and Meta-Analyses (PRISMA) guidelines and is registered with PROSPERO (CRD420251111460).

### 2.1. Data Source and Search Strategy

We conducted a systematic search in PubMed and Embase for studies published between January 2004 and October 2024, using the following search terms: “(‘electronic cigarette’ OR ‘e-cig*’ OR ‘e cig*’ OR ‘ecig*’ OR ‘electronic nicotine delivery’ OR ‘ENDS’ OR ‘vaping’ OR ‘vape*’ OR ‘vaper*’) AND (‘passive’ OR ‘secondhand’ OR ‘second hand’ OR ‘exposure’ OR ‘exposed’ OR ‘nonuser*’ OR ‘non-user*’)”. A total of 12,885 studies were retrieved; 5427 studies through PubMed and 7458 studies through EMBASE.

### 2.2. Eligibility Criteria

Eligible studies included peer-reviewed published randomized or non-randomized-controlled trials, retrospective or prospective cohort studies, observational studies, case series, and case reports that reported participants’ demographic information, underlying disease(s), and comorbidities, specifically secondhand exposure to e-cigarette smoke.

The outcome of interest in this study was the difference in health outcomes between participants exposed and unexposed to e-cigarettes. Health outcomes can be presented as clinical symptoms of respiratory and other body systems, or significant changes in laboratory investigations, biomarkers, or chemical substances that are related to organ functions. The exposure group included individuals who were directly exposed to e-cigarette smoke for a certain period of time, depending on each study.

Exclusion criteria included studies without human participants, studies with lone participants of firsthand smoking, studies without information on e-cigarette smoke, studies that include fetal exposure to maternal smoking, studies funded or supported by the e-cigarette or tobacco industry, and studies for which the full text could not be retrieved. Duplicate studies were taken into consideration. There were no language restrictions.

### 2.3. Data Extraction and Quality Assessment

TC, PP, YK, and SV independently screened the abstract or the full text of all 8635 studies retrieved from our search. Studies with uncertainties and disagreements in the final decision were resolved through discussion and voting among all authors. For each included study, the following information was extracted: first author, publication year, country of study, study design, sample size, age range of participants, body system, health outcome measures, summary of findings, and limitations (Table 1). We evaluated the quality of studies using the Newcastle–Ottawa Scale (NOS). The maximum score for the NOS’s 3 criteria (patient selection, comparability, and outcome evaluation) was 9 points, with studies scoring 7 or higher being deemed high quality.

## 3. Results

### 3.1. Study Characteristics

**Figure 1:**
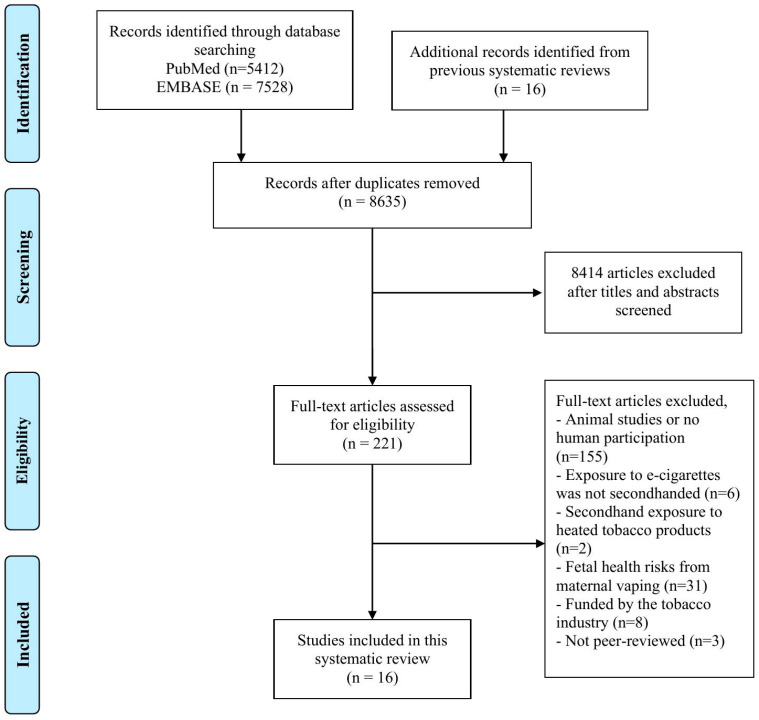
PRISMA diagram.

From the PRISMA diagram in Figure 1, a total of 8635 unique studies were retrieved, and after applying the eligibility criteria, 221 studies remained for full-text assessment. One hundred and fifty-five studies were subsequently excluded due to a lack of human participation, six were excluded due to first- or third-hand exposure, two were excluded due to exposure to heated tobacco products, thirty-one were excluded due to fetal exposure from maternal smoking, eight were excluded because they were funded by the tobacco industry, and three were excluded because of a lack of peer review. In the end, 16 studies fulfilled the criteria and were included in this systematic review [13,14,15,16,17,18,19,20,21,22,23,24,25,26,27,28].

From these sixteen studies [13,14,15,16,17,18,19,20,21,22,23,24,25,26,27,28] (Table 1), six [20,22,23,24,25,26] were conducted in the US, three [14,15,28] were from Greece, three [13,17,19] were from Spain, one [16] was from Kuwait, one [21] was from Italy, one [27] was from Denmark, and one [18] was conducted collaboratively across four countries (namely, Greece, Italy, Spain, and the United Kingdom). The study designs of the sixteen studies [13,14,15,16,17,18,19,20,21,22,23,24,25,26,27,28] included in our review were a case study [19], a cohort [23], eight experimental [14,15,17,18,25,26,27,28], four cross-sectional studies [16,20,22,26], and two observational studies [13,21]. Health outcome measures were self-reported health symptoms and biomarkers. Ten out of sixteen studies examined respiratory health effects [14,16,17,20,21,23,24,26,27,28], two examined cardiovascular health effects [25,26], one explored mental health effects [22], and six described biomarker exposure [13,14,15,17,18,19].

### 3.2. Respiratory Health Effects

Respiratory health risks due to SHA were self-reported health symptoms, such as bronchitis, shortness of breath, asthma attacks, throat irritations, and ear infections. In addition to self-reported symptoms, some studies measured lung function, breathing frequency, or blood oxygen levels. The first study related to respiratory health included in our review was reported in 2013. Flouris et al. [14] assessed the lung functions of 15 healthy never-smokers (aged 18–57 years) exposed to a passive e-cigarette smoking session (adjusted to simulate bar/restaurant levels) for 1 h. They concluded that a brief session of passive e-cigarette smoking resulted in a small (2.3%) drop in FEV1/FVC, indicating no major impact on normal lung function. In 2019, Bayly et al. [20] analyzed the 2016 Florida Youth Tobacco survey and concluded that youth (aged 11–17 years) who were in the same room or car with someone who had used e-cigarettes in the past 30 days had higher odds of reporting an asthma attack in the past 12 months after controlling for other relevant factors (adjusted OR, 1.27; 95% CI, 1.11–1.47). In 2020, Alnajem et al. [16] conducted a cross-sectional study of 1565 high school students in Kuwait and reported that frequent exposure to household SHA was linked to increased wheezing (aPR = 1.30, 95% CI 1.04–1.59), asthma (aPR = 1.56, 95% CI 1.13–2.16), and uncontrolled asthma symptoms (aPR = 1.88, 95% CI 1.35–2.62) compared to students who were not exposed. In addition, Tzortzi et al. [28] conducted an experimental study among 40 healthy non-smoking adults (aged 18–35 years) by having them experience SHA for 30 min in a closed 35 m^3^ room. Under this unrealistic exposure, they found a significant increase in fine and ultrafine particles (PM_1.0_ and PM_2.5_) concentrations, and participants reported a significant increase in ocular (itchiness, burning, watery eyes, and dryness), nasal (nasal drip, itchiness, dryness, sneezing, and stuffiness), throat-respiratory (dryness, soreness, cough, phlegm, and breathlessness) irritations after the experimental sessions. In 2021, McClelland et al. [26] conducted an experimental study in which 76 individuals used e-cigarettes next to 73 (aged 18–63 years) who had never used e-cigarettes for 20 min in a 12 ft^3^ enclosed room. They found that immediate exposure to e-cigarettes caused significant increases in oral temperature, but did not affect breathing frequency or blood oxygen levels. Rosenkilde et al. [27] conducted another experimental study among 16 non-smoking chronic obstructive pulmonary disease (COPD) patients who were randomly exposed to SHA for 4 h and clean air. Under an unrealistic condition, they found that SHA exposure had a negative effect on Surfactant Protein-A (SP-A) and some plasma proteins, indicating respiratory inflammation, and participants reported experiencing throat irritation during the session. In 2022, Islam et al. [23] used longitudinal data from 2014–2019 to examine whether exposure to SHA at home was associated with adverse respiratory health symptoms among young adults. They found that after controlling for relevant risk factors, SHA exposure was associated with bronchitic symptoms (OR 1.40, 95% CI 1.06 to 1.84) and shortness of breath (OR 1.53, 95% CI 1.06 to 2.21). In 2024, Costantino et al. [21] conducted a retrospective study of 54 children (aged 5–17 years) with asthma and found that those who were exposed to SHA from parents at home experienced higher exacerbations, and required more rescue therapy and therapeutic step-ups; however, all findings were not statistically significant due to the small sample size. Also, Lam et al. [24] found that SHA exposure was associated with self-reported ear infections (OR = 1.61, 95% CI 1.01–2.58).

### 3.3. Cardiovascular Health Effects

Lee et al. (2019) [25] conducted an experimental study on five healthy non-smokers, exposing them to SHA for two 1 h sessions over two days, and measured cardiac autonomic response to nicotine exposure. They found that short-term SHA exposure, as determined by nicotine concentrations, was linked to both a reduction in heart rate variability (HRV) and a shortening of the heart rate-corrected QT (QTc) interval after adjusting for potential covariates such as age and body mass index (BMI) [25]. McClelland et at (2021) [26] collected physiological data from nonsmokers who were immediately exposed to SHA, including two cardiovascular-related parameters (blood pressure and heart rate). In contrast to e-cigarette user groups, they did not see elevated blood pressure and heart rates in non-vaping participants exposed to SHA [26].

### 3.4. Mental Health Effects

Farrell et al. (2022) [22] used the fourth wave of the Population Assessment of Tobacco and Health (PATH) Study, a nationally representative study of U.S. adults, to examine whether SHA exposure was linked to a higher risk of internalizing mental health disorders by the Global Appraisal of Individual Needs–Short Screener (GAIN–SS) across four items: (1) depression include feeling trapped, lonely, sad, blue, depressed, or hopeless about the future; (2) sleep disturbances such as bad dreams, restlessness, or falling asleep during the day; (3) anxiety, nervousness, tenseness, fear, panic, or a sense of impending doom; and (4) distress when reminded of the past. Participants who had experienced at least two of the four items were considered to have moderate-to-severe internalizing mental health disorders [22]. They found that non-users who were exposed to SHA had a higher risk of experiencing moderate-to-severe internalizing mental health disorders, compared to those who were not exposed (AOR = 1.43, CI 1.03–1.99) [22].

### 3.5. Biomarkers of Exposure

Several studies on SHA exposure measured relevant biomarkers, including byproducts of nicotine metabolism such as cotinine levels. Flouris et al. (2012, 2013) [14,15] found no significant changes in complete blood count (CBC) indices among never-smokers exposed to SHA in a closed 60 m^3^ chamber for 1 h, but their serum cotinine levels were higher immediately after a passive e-cigarette exposure session compared to participants in the control group. Ballbè et al. (2014) [13] found a significant increase in cotinine levels in saliva and urine among non-smokers living with nicotine e-cigarette users at home compared to non-smokers from control homes. They also found that the cotinine levels did not differ between non-smokers living in conventional cigarette-smoking homes and e-cigarette use homes [13]. Similarly, another study conducted in four European countries measured e-cigarette aerosol-related biomarkers in the saliva and urine samples of non-users in e-cigarette users’ homes (those with a daily e-cigarette user who used e-cigarettes for at least 1 month prior to the study) compared to control homes where no one used e-cigarettes or any tobacco products [18]. This home setting experiment was more realistic when compared to other experimental studies, and measurements of biomarkers were more robust than self-reported questionnaires. The researchers found slightly higher but comparable levels of cotinine, 3′-OH-cotinine, and 1,2-propanediol in the saliva of non-users living in exposed homes than non-users living in control homes, although the levels were significantly lower than in e-cigarette users [18]. Ballbè et al. (2023) [19] conducted a 12-week prospective longitudinal case study (BERNAT study) in a family unit (a 130-m^3^ flat) involving an e-cigarette user, his pregnant wife, and their son to assess SHA exposure in a home setting. They found a substantial increase in nicotine metabolites in the non-smoking wife’s urine, hair, saliva, and breast milk, and the 3-year-old child’s urine and hair, both of whom were exposed to SHA from e-cigarettes for an average of 7–8 h per day on weekdays and 14–15 h per day during weekends [19]. They also detected low levels of metals in both the pregnant mother and the child [19].

## 4. Discussion

Our systematic review emphasizes that exposure to SHA is not harmless and may have negative health consequences. Research studies on the effects of SHA included in this study are related to the respiratory system, cardiovascular system, mental health, and biomarkers. The most common self-reported SHA symptom was respiratory health, such as bronchitic symptoms, shortness of breath, and asthma. The respiratory system is generally the first to experience the immediate impact of involuntary vaping or smoking because it directly enters and irritates the respiratory airways and lungs.

Several studies found that levels of fine particulate matter (PM_2.5_) and ultrafine particles (UFPs) increased after e-cigarette use, both under real-world conditions [29] and in experimental chambers [12]. For example, a study found that levels of PM_2.5_ increased 160-fold at a distance of 0.5 m and 103-fold at 1 m from an e-cigarette user in a closed room [29]. Another study also found that PM_2.5_ concentrations were higher during vape store opening hours than during closing hours, and the concentrations were correlated to the number of e-cigarette users present [30]. However, these studies were conducted in a non-natural context, and the exposures may be unrealistic. Recent systematic reviews reported that the average particle mass concentrations from e-cigarette users were comparable to combustible cigarette smoking in indoor environments [31,32]. However, particle number concentrations of secondhand smoke (SHS) are generally higher than those of SHA [32], and their chemical constituents are different [33]. Although no current evidence indicates that particular components derived from SHA cause health effects (as no combustion process is involved) further investigations are needed to explore this issue.

E-cigarette aerosols generally have lower concentrations of chemical components than combustible cigarettes, and unlike SHS, there is no side-stream emission in SHA [31,34]. However, levels of nicotine, vegetable glycerin, vaporized propylene glycol, and hazardous chemicals like aldehydes and heavy metals of SHA have been well documented, although these compounds were classified at levels below SHS [31,32] and are comparable to non-smoking environments without vaping [35]. Some studies reported that about 90% of the inhaled aerosol was retained; thus, the exhaled aerosols from e-cigarettes were strongly diluted [36]. However, it should be noted that this figure depended on users’ vaping behaviors because some outlier users could exhale as much as 51% of the inhaled nicotine and generate significant SHA [36].

Environmental parameters such as PM and airborne density were nearly identical in vaping and non-vaping homes [18]; the discernible difference between passively exposed non-users and controls was biomarkers indicating significantly higher levels of glycerol and propylene, as well as slightly higher cotinine levels [18,35]. Biomarker measures varied among studies, except for cotinine concentrations in non-smokers/vapers exposed to secondhand e-cigarettes, which were likely to be higher than in non-exposed individuals. Several studies demonstrated that urine cotinine concentrations in e-cigarette users were comparable to those in smokers [37,38]. Our review also found that levels of urine cotinine among non-smokers exposed to cigarettes and e-cigarettes were statistically similar [13]. Furthermore, the cotinine levels in the urine and saliva of non-users exposed to e-cigarettes in the most reliable experimental study in our review [18] were comparable to those of passive smokers in earlier studies but remained well below levels in e-cigarette users [39,40]. Elevated cotinine levels among passive smokers are associated with the risk of several diseases, such as coronary heart disease [41] and respiratory diseases, especially among children [42,43]. Nevertheless, levels of cotinine among non-users exposed to e-cigarettes may be influenced by occasional exposure to environmental smoke, as the levels found are in the same range as those of non-smokers [44].

Studies found that the concentration of nicotine in e-cigarettes continues to rise, especially since disposable e-cigarettes entered the market [45,46]. In 2015, when JUUL entered the market, the packaging of a JUUL pod 5% strength cartridge indicated that it was ‘approximately equivalent to about 1 pack of cigarettes’ [47]. However, by 2022, at least 48 new e-cigarette products with 6% nicotine strength (a 20% increase compared to the JUUL pod at 5%) and at least 18 items had a 20 mL capacity, 29 times the size of one JUUL refill [48]. However, all studies in our review that measured cotinine concentration were conducted before 2019, when newly disposable e-cigarettes were not available. Therefore, further research is needed to explore whether cotinine levels among e-cigarette users and those exposed to SHA increase as the concentration of nicotine in e-cigarettes continues to soar.

Evidence for the risk of SHA is essential to policy development to protect the health of the public. The 2025 WHO report on the global tobacco epidemic revealed that the number of countries adopting or expanding smoke-free laws to cover e-cigarettes continued to rise [49]. In 2024, 99 countries either completely or partially banned e-cigarette use in public places, compared to 84 countries in 2020 [49]. Despite progress in prohibiting e-cigarette use in public areas, e-cigarette use at home is also critical and needs to be addressed and controlled. Compared to smokers, e-cigarette users are more likely to use their devices indoors, especially at home [50].

Studies in our review encountered potential limitations. Firstly, most health-related symptoms were self-reported. Secondly, 11 out of 16 studies had sample sizes lower than 100. Studies with larger sample sizes were cross-sectional studies, which were unable to measure a causal relationship. Thirdly, the duration of SHA exposure was extremely short, with some sessions as minimal as 20 min [26]. Although these experiments were short, they were able to find some interesting health outcomes, such as respiratory symptoms. However, most experimental studies were conducted under unrealistic conditions as the number of puffs during the experiment were much higher than in normal environments [17,27,28]; therefore, their outcomes may be unreliable. Also, some studies relied on non-probability sampling. Further research is needed to explore the health effects of SHA over a longer period in a more realistic environment. Despite these limitations, our study has explored and summarized the health effects of SHA exposure on humans, which are relevant to the public and policymakers.

## 5. Conclusions

Our review found that secondhand e-cigarette exposure is not harmless and may have negative health consequences. The most significant biomarker found in non-smokers/vapers exposed to secondhand e-cigarettes was cotinine concentrations, which were comparable to those of passive smokers. Among self-reported health consequences, respiratory health was the most prominent. Nevertheless, higher-quality prospective studies remain essential to examine long-term secondhand exposure.

## Figures and Tables

**Table 1:** Summary of studies included in the systematic review.

First Author (Publication Year)	Country of Study	Study Design	Sample Size	Age of Participants	System	Health Outcome Measures	Summary of Findings	Limitations
Alnajem A, et al. (2020) [16]	Kuwait	Cross-sectional study	1565	16–19	Respiratory	Self-reported asthma symptoms	The frequency of exposure to household SHA from e-cigarettes was associated with asthma symptoms. For example, compared to those with no exposure to household SHA, frequent exposure to household SHA was associated with current wheeze (aPR = 1.30, 95% CI 1.04–1.59), current asthma (aPR = 1.56, 95% CI 1.13–2.16), and current uncontrolled asthma symptoms (aPR = 1.88, 95% CI 1.35–2.62).	Self-reported, and the inability to assess temporal sequence of events
Amalia B, et al. (2021) [17]	Spain	Experimental study	2	40–49	Respiratory, Immunology	Biomarkers included saliva nicotine, cotinine, 3-OH-cotinine, nornicotine, NNN, NNK, NNAL, 1,2-PG, 1,3-PG, and glycerol, and short-term health symptoms: irritation relating to ocular (itchiness, burning, watery eyes and dryness), nasal (nasal drip, itchiness, dryness, sneezing and stuffiness), throat-respiratory (dryness, soreness, cough, phlegm and breathlessness) and general complaints (headache, nausea and fatigue)	All biomarkers collected from nonvaping exposed to e-cigarettes were below the limit of quantification; however, short-term irritation symptoms, including dry throat, nose, eyes, and phlegm were reported.	Self-reported; short duration of exposure, and unrealistic exposure conditions
Amalia B, et al. (2023) [18]	Greece, Italy, Spain, and the United Kingdom	Experimental study	50	30–49	Immunology	Biomarkers included saliva nicotine, cotinine, 3-OH-cotinine, nornicotine, NNN, NNK, NNAL, 1,2-PG, 1,3-PG, and glycerol, and self-reported overall health status	Non-users residing with e-cigarette users had low but significantly higher levels of cotinine, 3′-OH-cotinine, and 1,2-propanediol in saliva than non-users living in control homes	Convenience sampling of participants
Ballbè M, et al. (2014) [13]	Spain	Observational study	54	n/a	Immunology	Saliva and urine cotinine	Cotinine concentrations in non-smokers exposed to e-cigarette vapour at home were significantly higher than in non-smokers from control homes	Convenience sampling of participants and small sample size; only 5 participants in the e-cigarette exposure group
Ballbè M, et al. (2023) [19]	Spain	Case study	3	3, 40, 47	Immunology	Urine, saliva, hair, cord blood and breast milk for nicotine and metabolites (cotinine, 3 OH-cotinine, nornicotine), TSNAs (NNN, NNK, NNAL), 1,2-PD and 1,3-PD, and glycerol	Nicotine and its metabolites were found in a child exposed to e-cigarettes from parents at home.	Small sample size and short duration of exposure; realistic condition, but of very limited scope
Bayly JE, et al. (2018) [20]	United States	Cross-sectional study	11,830	11–17	Respiratory	Self-reported asthma attack	Secondhand e-cigarette exposure was associated with higher odds of reporting an asthma attack in the past 12 months (adjusted OR, 1.27; 95% CI, 1.11–1.47)	Self-reported, and the inability to assess the temporal sequence of events
Costantino S, et al. (2024) [21]	Italy	Observational study	54	5–17	Respiratory	Asthma exacerbations, need for rescue therapy, need for therapeutic step-up, ACT/c-ACT score	Asthmatic patients exposed to secondhand e-cigarettes at home had higher exacerabtions, needed more rescure therapy, therapeutic step-up, and ACT/c-ACT score; however, all results were not statistically significant	Small sample size and self-reported symptoms
Farrell KR, et al. (2022) [22]	United States	Cross-sectional study	16,173	18 and older	Mental health	Internalizing mental health disorders	Secondhand e-cigarette emissions exposure among non-users (AOR = 1.43, 1.03–1.99) was associated with increased odds of moderate-to-severe internalizing mental health problems compared to unexposed non-users	Inability to assess the temporal sequence of events
Flouris AD, et al. (2012) [15]	Greece	Experimental study	15	28.87	Immunology	CBC	CBC indices remained unchanged	Small sample size, short duration of exposure
Flouris AD, et al. (2013) [14]	Greece	Experimental study	15	18–57	Respiratory, Immunology	Serum cotinine, lung function (FVC, FEV1, FEV1/FVC ratio, PEF, FVC (FEF25-75)), exhaled CO, FeNO	Serum cotinine was significantly increased after a passive e-cigarette smoking session (silimar to tobacco cigarette smoking); however, lung function, CO, and FeNo remained unchanged compared with a control session.	Small sample size, short duration of exposure
Islam T, et al. (2022) [23]	United States	Cohort study	2097	17.3 (first year), and 21.9 (the follow-up year)	Respiratory	Self-reported bronchitic symptoms, wheezing, and shortness of breath	After additionally adjusting for secondhand smoking, secondhand cannabis, and primary vaping or smoking, the secondhand e-cigarette exposure was associated with bronchitic symptoms (OR 1.40, 95% CI1.06 to 1.84) and shortness of breath (OR 1.53, 95% CI 1.06 to 2.21); however, the association between secondhand vaping and wheezing was not statistically significant.	Measurement error due to self-reported exposure and respiratory outcomes
Lam TK, et al. (2023) [24]	United States	Cross-sectional study	2022	6–17	ENT	Self-reported ear infections	E-cigarette exposure in the last 7 days was associated with ≥3 ear infections (OR = 1.61, 95% CI 1.01–2.58, *p* = 0.047)	Self-reported, and the inability to assess the temporal sequence of events
Lee, et al. (2019) [25]	United States	Experimental study	5	29.4	Cardiovascular	Heart rate-corrected QT interval	Decreased HRV and shortening of the QTc were found during short-term secondhand exposures to e-cigarette emissions	Small sample size, short duration of exposure
McClelland ML, et al. (2021) [26]	United States	Experimental study	73	18–63	Cardiovascular, Respiratory	Immediate physiological effects (BP, mean arterial pressure, RR, FVC, blood sugar, SpO2, oral temperature)	Oral temperatures of nonvaping participants were significantly higher after passive exposure to e-cigarettes; however, other measures were not significantly affected.	Short duration of exposure
Rosenkilde K, et al. (2021) [27]	Denmark	Experimental study	16	56–77	Respiratory	SP-A and albumin in exhaled air, FEV1, FVC, FEV1/FVC, FeNO, plasma proteins, and self-reported symptoms	Exposure to vapes reduced SP-A in exhaled air while increasing numerous plasma proteins considerably. Throat discomfort increased following passive vape exposure, whereas FVC and FEV1 decreased, but not significantly.	Small sample size, short duration of exposure, unrealistic exposure conditions, and not all participants completed all health examination
Tzortzi A, et al. (2020) [28]	Greece	Experimental study	40	18–35	ENT, Respiratory	Symptoms of irritation relating to ocular (itchiness, burning, watery eyes and dryness), nasal (nasal drip, itchiness, dryness, sneezing and stuffiness), throat-respiratory (dryness, soreness, cough, phlegm and breathlessness) and general complaints (headache, nausea and fatigue)	Ocular, nasal, throat-respiratory symptoms and general complaints also increased significantly and lasted up to 30 min after the exposure and were positively associated with the concentrations of the VOC mixture emitted	Self-reported; short duration of exposure, and unrealistic exposure conditions

## Data Availability

All data used to prepare this paper are available from the cited sources.
